# Targeting Interleukin(IL)-30/IL-27p28 signaling in cancer stem-like cells and host environment synergistically inhibits prostate cancer growth and improves survival

**DOI:** 10.1186/s40425-019-0668-z

**Published:** 2019-07-31

**Authors:** Carlo Sorrentino, Zhinan Yin, Stefania Ciummo, Paola Lanuti, Li-Fan Lu, Marco Marchisio, Matteo Bellone, Emma Di Carlo

**Affiliations:** 1 Department of Medicine and Sciences of Aging, G. d’Annunzio University of Chieti-Pescara, Via L. Polacchi 11, 66100 Chieti, Italy; 2 Anatomic Pathology and Immuno-Oncology Unit, Center for Advanced Studies and Technology (CAST), G. d’Annunzio University of Chieti-Pescara, Chieti, Italy; 3 The First Affiliated Hospital, Biomedical Translational Research Institute, Guangdong Province Key Laboratory of Molecular Immunology and Antibody Engineering, Jinan University, Guangzhou, China; 4 Division of Biological Sciences, Center for Microbiome Innovation and Moores Cancer Center, University of California, San Diego, La Jolla, CA USA; 5 Cellular Immunology Unit, San Raffaele Scientific Institute, Milan, Italy

**Keywords:** Interleukin-30, Tumor microenvironment, Prostate Cancer Stem-Like Cells, Treg cells, Immunohistochemistry

## Abstract

**Background:**

Interleukin(IL)-30/IL-27p28 production by Prostate Cancer (PC) Stem-Like Cells (SLCs) has proven, in murine models, to be critical to tumor onset and progression. In PC patients, IL-30 expression by leukocytes infiltrating PC and draining lymph nodes correlates with advanced disease grade and stage. Here, we set out to dissect the role of host immune cell-derived IL-30 in PC growth and patient outcome.

**Methods:**

PC-SLCs were implanted in wild type (WT) and IL-30 conditional knockout (IL-30KO) mice. Histopathological and cytofluorimetric analyses of murine tumors and lymphoid tissues prompted analyses of patients’ PC samples and follow-ups.

**Results:**

Implantation of PC-SLCs in IL-30KO mice, gave rise to slow growing tumors characterized by apoptotic events associated with CD4^+^T lymphocyte infiltrates and lack of CD4^+^Foxp3^+^ T regulatory cells (Tregs). IL-30 knockdown in PC-SLCs reduced cancer cell proliferation, vascularization and intra-tumoral Indoleamine 2,3-Dioxygenase (IDO)^+^CD11b^+^Gr-1^+^ myeloid-derived cells (MDCs) and led to a significant delay in tumor growth and increase in survival. IL-30-silenced tumors developed in IL-30KO mice, IL-30^−/−^tumors, lacked vascular supply and displayed frequent apoptotic cancer cells entrapped by perforin^+^TRAIL^+^CD3^+^Tlymphocytes, most of which had a CD4^+^T phenotype, whereas IL-10^+^TGFβ^+^Foxp3^+^Tregs were lacking. IL-30 silencing in PC-SLCs prevented lung metastasis in 73% of tumor-bearing WT mice and up to 80% in tumor-bearing IL-30KO mice.

In patients with high-grade and locally advanced PC, those with IL-30^−/−^tumors, showed distinct intra-tumoral cytotoxic granule-associated RNA binding protein (TIA-1)^+^CD4^+^Tlymphocyte infiltrate, rare Foxp3^+^Tregs and a lower biochemical recurrence rate compared to patients with IL-30^+/+^tumors in which IL-30 is expressed in both tumor cells and infiltrating leukocytes.

**Conclusion:**

The lack of host leukocyte-derived IL-30 inhibits Tregs expansion, promotes intra-tumoral infiltration of CD4^+^T lymphocytes and cancer cell apoptosis. Concomitant lack of MDC influx, obtained by IL-30 silencing in PC-SLCs, boosts cytotoxic T lymphocyte activation and cancer cell apoptosis resulting in a synergistic tumor suppression with the prospective benefit of better survival for patients with advanced disease.

**Electronic supplementary material:**

The online version of this article (10.1186/s40425-019-0668-z) contains supplementary material, which is available to authorized users.

## Background

Prostate cancer (PC) is the most common non-cutaneous malignant neoplasm in men [1]. As its incidence increases with age, an increase in the number of new cases is expected in the near future due to the aging of the population [2]. Mortality for PC is mainly due to metastatic disease, for which there is no effective cure. The definition of the molecular mechanisms driving this process is crucial to identify the right therapeutic targets.

Originally identified as the p28 subunit of the heterodimeric cytokine Interleukin(IL)-27 [3, 4], and found to suppress the antitumor effects of IL-27 in colon cancer [5], IL-30 is emerging as a new and intriguing factor that may condition PC onset and progression [6–8]. It is produced by tumor-infiltrating leukocytes, mostly myeloid derived cells (MDCs), in approximately 77% of metastatic PC. Expression of IL-30 in PC and leukocytes infiltrating tumor and draining lymph nodes is associated with poorly differentiated high grade and stage of disease [6]. High levels of IL-30 in MDCs of tumor-draining lymph nodes from breast cancer patients have also proven to be an independent predictor of poor prognosis [9], thus suggesting the involvement of IL-30, produced by the host’s immune cells, in conditioning tumor behavior and patient outcome.

Our most recent study revealed that PC Stem-Like Cells (PC-SLCs), which are believed to be responsible for tumor initiation, progression and treatment resistance [10, 11], are a significant source of IL-30 in PC [8]. IL-30 promotes PC-SLC viability, self-renewal ability, tumorigenic and metastatic potential, as revealed by using a fully immunocompetent orthotopic murine model and it regulates, essentially via STAT1 and STAT3 signaling, a range of pro-inflammatory and chemokine/chemokine-receptor genes that promote tumor growth [8]. IL-30 knockdown in PC-SLCs hinders their engraftment and dramatically compromises tumor onset and progression. However, beyond IL-30 produced by cancer cells and acting in autocrine and paracrine loops, the function of endogenous IL-30 released by tumor- and draining lymph node-infiltrating leukocytes (ILK), which has been associated with a worse prognosis, remains to be addressed.

In this study, IL-30/IL-27p28 conditional knockout mice were used as recipients for PC-SLC implantation and tumor growth to determine whether host immune cell-derived IL-30 is essential to PC progression and therefore if it should be considered in planning an effective cytokine targeted immunotherapy to treat or prevent the metastatic disease.

## Methods

### Cell cultures

Murine Prostatic Intraepithelial Neoplasia (PIN)-derived Stem-Like Cells (PIN-SCs) were isolated from B6 TRAMP mice [12] and characterized in refs. [13, 14].

For our purposes, we used, in addition to wild type PIN-SCs, two of the cell lines stably silenced for the IL-30 gene (by using short hairpin, sh, RNA Hush GFP-tagged lentiviral vectors from Origene), which exhibited the highest knockdown efficiency (IL-30shPIN-SCs clone D: 89% and clone B: 82%), and related control cells transfected with non-effective scrambled shRNA, namely shPIN-SCs, generated in our laboratory and previously described [8]. Cell lines were authenticated by means of cell surface staining for characteristic markers, as described [13, 14], by in vitro tests (cell proliferation and sphere formation) and ELISA assay for IL-30 (mIL-27p28/IL-30 Quantikine ELISA kit, R&D) as described [8]. Since the two IL-30-silenced cell lines we used demonstrated similar biological behavior in vitro [8] and in vivo, only results from Clone D are shown.

Cells were cultured using serum-free medium (SFM), which consisted of DMEM:F12 (1:1), GlutaMAX-I supplement (Invitrogen), 50 ng/ml heparin (Sigma-Aldrich), 20 ng/ml EGF, and 10 ng/ml βFGF (R&D), as described [14]. Mycoplasma contamination was excluded by using the MycoAlert™ PLUS Mycoplasma Detection Kit (Lonza).

### Mouse studies

The IL-27p28 conditional knockout mouse strain (EIIa-p28^f/f^), that has been described by *Zhang* et al. [15], was kindly provided by Prof. Yin Z. (Jinan University, Guangdong, China) and Prof. Lu L.F. (University of California, San Diego, CA, USA), whereas Wild Type C57BL/6 J (WT) mice were purchased from Envigo. The genotyping of EIIa-p28^f/f^ mice was performed via polymerase chain reaction (PCR), using the following primers (Sigma-Aldrich): TCCCTTCCAGGACCATACTGCTAA (forward) and ACCCAAACACAGGCCAGTACTCTA (reverse) to detect the WT band (252 bp); CTGCAGCCAAGCTATCGAATTCCT (forward) and TGCATCACCACACTTGGCGTACTA (reverse) to detect the null band (230 bp). The PCR procedures were carried out on an MJ Mini Gradient Thermal Cycler (Bio-Rad) under the following conditions: 95 °C for 4 min, followed by 35 amplification cycles (denaturation at 94 °C for 45 s, annealing at 66 °C for 45 s and extension at 72 °C for 1 min). The PCR products were separated on a 2% agarose gel stained with ethidium bromide and the DNA bands were visualized with a Transilluminator 2000 (Bio-Rad). The p28 knockout efficiency was confirmed by quantitative real-time PCR, on peripheral blood leukocytes, and by ELISA, on serum samples, using the mIL-27p28/IL-30 Quantikine ELISA kit (R&D; detection sensitivity 4.27 pg/ml) according to manufacturer’s instructions.

In addition to WT mice, we used B6 EIIa-cre mice (Jackson Laboratory) and p28^f/f^ mice (carrying two LoxP sites flanking *p28* exons 2, 3 and 4) as controls, because *Cre* expression and *p28* floxing could contribute, by themselves, to the mouse phenotype.

Groups of 45 mice were subcutaneously (sc) injected with 1X10^5^ PIN-SCs, shPIN-SCs or IL-30shPIN-SCs and monitored 2 times per week. Tumors were measured with calipers as soon as they were palpable and until evidence of suffering was observed. Then, mice were euthanized and tumors and other organs were collected for morphological and molecular analyses.

- Power calculation - Since a one-sided log rank test, with an overall sample size of 30 mice per group, achieves an 90% power, at a 0.05 significance level, to detect a difference of 30% in tumor growth, 30 mice per group were kept until evidence of suffering was observed. Fifteen mice, from each group, were sacrificed for histopathological studies, at key time points (groups of 3) based on tumor growth and progression rate.

### Flow cytometry

Spleens from WT or EIIa-p28^f/f^ mice (sc injected or not with PIN-SCs) were excised and cut into small pieces that were crushed through a Corning® cell strainer (size 40 μm) using a syringe plunger. Subsequently, the cells were resuspended in 2 ml of pre-warmed lysing solution (BD Biosciences) and incubated at 37 °C in a water bath for 2 min. To assess phenotype markers, PIN-SCs were harvested and mechanically dissociated into a single cell suspension.

Then, the cells were pelleted, resuspended in PBS and incubated for 30 min, at 4 °C, with the antibodies (Abs) listed in Additional file 1: Table S1 at a concentration of 0.25 μg/100 μl. Acquisition was performed using a BD FACSCanto II instrument and the data were analyzed using FlowJo software. Dead cells were excluded by 7AAD staining. All experiments were performed in triplicate.

### Patients and samples

Prostate tissue samples were obtained from patients who underwent radical prostatectomy for PC, between 2009 and 2013, at the S.S. Annunziata Hospital (Chieti, Italy). PC patients, ages 60–70, had not received immunosuppressive treatments, hormone- or radio-therapy [16] and were free from immune system diseases. They were followed-up for at least 5 years after prostatectomy.

Biochemical recurrence (BCR) was defined as a PSA value > 0.2 ng/ml after prostatectomy, confirmed by another measurement after 4 weeks [17].

Clinic-pathological stages were determined according to the seventh edition of the TNM classification of malignant tumors [18] and tumor grade was assessed according to the Gleason scoring system from the prostate biopsies [19]**.**

For this study, we analyzed n.112 PC samples obtained from patients at Stage III (pT3N0M0, with negative surgical margins), with Gleason score of 8–10. After staining for IL-30, we selected and then analyzed only PC specimens that were found (i) to express IL-30 in both PC cells and ILK (referred to as IL-30^Pos^PC; *n*. 25) or (ii) to lack IL-30 expression in both PC cells and ILK (referred to as IL-30^Neg^PC; *n*. 59), according to the criteria that we defined previously [6] and describe below.

IL-30 expression in neoplastic cells, of human PC specimens, was evaluated using the following criteria, that we previously applied [6] and that are based on 1) the widening of the staining expressed as the percentage of tumor stained, i.e.: < 50%, between 50 and 70%, and > 70%, and 2) the strength of the staining: defined as absent (−), slight (±), distinct (+) or strong (++).

Thus, IL-30 immunostaining was defined as:

- *positive,* when a) the widening was > 70% and its strength ranged from slight (±) to strong (++), or b) the widening was between 50 and 70% and its strength ranged from distinct (+) to strong (++);
- *weakly positive,* when a) the widening was between 50 and 70% and its strength was slight (±), or b) the widening was equal to 50% and its strength ranged from slight (±) to strong (++);
- *negative* when the widening was < 50% and its strength was slight (±) to absent (−).

ILK expression of IL-30 in human PC samples was evaluated using the following score, based on 1) the percentage of leukocyte expressing the cytokine, i.e. < 50%, between 50 and 70%, and > 70%, and 2) the strength of the cytokine staining, that was defined as absent (−), scarce (±), distinct (+) or strong (++).

Thus, IL-30 expression by ILK was defined as:

- *strong,* when a) the staining involved more than 70% of leukocytes and its strength ranged from scarce (±) to strong (++), or b) the percentage of positively stained leukocytes was between 50 and 70% and the strength of the staining ranged from distinct (+) to strong (++);
- *distinct,* when a) the staining involved > 50% and ≤ 70% of leukocytes and its strength was scarce (±), or b) the staining involved 50% of leukocytes and its strength ranged from scarce (±) to strong (++);
- *scanty,* when the staining involved < 50% of leukocytes and its strength ranged from scarce (±) to absent (−).

Therefore, PC samples with *positive* and *strong* IL-30 expression were classified as IL-30^Pos^PC, whereas PC samples with *negative* and *scanty* IL-30 expression were classified as IL-30^Neg^PC.

Immunostained sections were examined by two pathologists in a blind fashion, with very good agreement (κ value = 0.89 and 0.78 for evaluation of IL-30 staining in PC cells and ILK, respectively).

### Histopathology, immunohistochemistry, confocal microscopy and TUNEL staining

For histology and immunohistochemistry, human PC specimens and half of each murine tissue sample were fixed in 4% formalin, embedded in paraffin and sectioned at 4 μm for hematoxylin and eosin (H&E) or immunostaining. For double immunofluorescent stainings, the other half of the murine sample was embedded in Killik frozen section medium (Bio-Optica), snap frozen in liquid nitrogen and preserved at − 80 °C.

Single, double (CD11b/Gr-1, CD11b/IDO, IL-30**/**F4/80, Foxp3/CD4, Foxp3/IL-10, Foxp3/TGFβ, CD3/perforin, caspase-3 (Casp3)/CD3, CD4/TIA-1, F4/80**/**IFNγ and CD3/IFNγ) or triple (IL-30/CD11b/Gr-1) immunostainings on formalin-fixed, paraffin-embedded mouse and human tissue sections, were performed using the Abs listed in Additional file 1: Table S2, as reported [6, 8, 20].

Double immunofluorescent stainings (CD3/TRAIL and CD4/FasL) were performed on frozen sections, as described [20], and examined using a Zeiss LSM 510 Meta laser scanning confocal microscope (Zeiss).

Since formaldehyde denatures tissue macromolecules, thus making some tissue antigens inaccessible to the primary Abs (antigen masking), when necessary, we performed “antigen unmasking” by heat induced epitope retrieval. Sensitivity and specificity of the stainings were confirmed by testing serial dilutions of each Ab on appropriate positive control tissues, listed in Additional file 1: Table S2, whereas sections incubated with non-immune sera or diluent were used as negative controls. Rodent Block (Biocare Medical) was used to minimize endogenous Ig staining when using mouse primary Abs on mouse tissue.

TUNEL staining was performed with the ApopTag Peroxidase In Situ Apoptosis Detection Kit (Merck Millipore) following manufacturer’s instructions, using spleen sections as positive control.

Proliferation index, microvessel and cell counts were assessed by light microscopy, at × 400, in an 85431.59 μm^2^ field, on single immunostained sections, with Qwin image analysis software (version 2.7), which has the following highly reproducible steps: 1) image acquisition; 2) conversion of RGB image (true colors) to binary image (black and white); 3) filtering to remove noise; 4) counting of immunostained cells or measurement of positively stained area.

For morphometric analyses on mouse tissue samples, six-eight high-power fields were evaluated for each section and three sections per sample were analyzed. Results are expressed as mean ± SD of positive cells per field (F4/80, CD11b/Gr-1, Foxp3/CD4, CD3, NKp46, Ly-6G, CD4, CD8) or mean percentage of positive cells/number of total cells (Sca-1, PCNA, TUNEL, caspase-3, perforin).

CD4^+^ and Foxp3^+^ cell counts, on human PC samples, were performed by adding together the positive cells scattered in fields randomly chosen within neoplastic areas and values are represented as the mean ± SD of positive cells/field. Eight to 12 high-power fields were examined for each section and 2 sections per sample were evaluated, as reported [20].

### Statistical analysis

For in vitro and in vivo studies, between-group differences were assessed by Student’s *t*-test and ANOVA (followed by Tukey HSD test). Between-group differences in sphere-forming potential were evaluated by ELDA (*Hu* et al. *J Immunol Methods 2009)*. Survival curves were constructed using the Kaplan-Meier method and survival differences were analyzed by the log-rank test. Follow-up time was 60 months. All statistical tests were evaluated at an α level of 0.05, using Stata version 13 (Stata Corp).

## Results

### The lack of IL-30 in PC-SLCs and host environment synergistically inhibits tumor growth, reduces lung metastasis and prolongs host survival

**Fig. 1:**
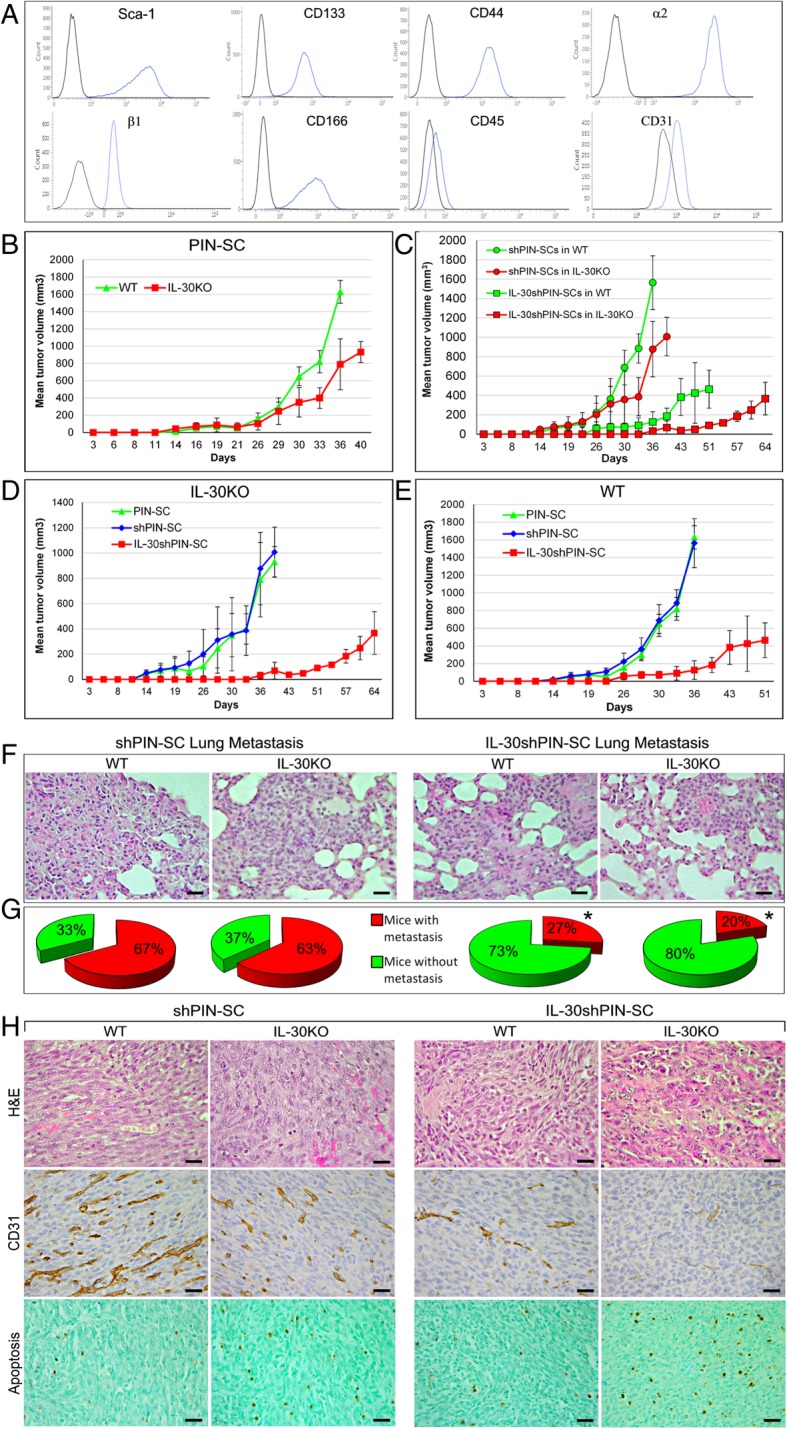
Characteristics of the growth and progression of tumors developed after sc implantation of IL-30-silenced PIN-SCs in WT and IL-30KO mice. **a** Flow cytometric profiling of phenotype markers expressed by PIN-SCs. Blue profiles illustrate the expression of specific markers, while black profiles represent isotype controls. Each panel is representative of three independent experiments. **b** Mean volume of tumors developed after implantation of PIN-SCs in WT or in IL-30KO mice. Student’s t-test: *p* < 0.0001 versus WT mice. Results from B6 EIIa-cre mice and p28^f/f^ mice are not different from those obtained in WT mice (Fisher Exact Probability Test: *p* > 0.99). **c** Mean volume of tumors developed after implantation of shPIN-SCs or IL-30shPIN-SCs in WT or in IL-30KO mice. Student’s t-test: *p* < 0.001 (shPIN-SCs or IL-30shPIN-SCs in IL-30KO mice versus WT mice). Results from B6 EIIa-cre mice and p28^f/f^ mice are not different from those obtained in WT mice (Fisher Exact Probability Test: *p* > 0.99). **d** Mean volume of tumors developed after implantation of PIN-SCs, shPIN-SCs or IL-30shPIN-SCs in IL-30KO mice**.** ANOVA: *p* < 0.001. Tukey’s HSD test: *p* < 0.01 versus both controls. **e** Mean volume of tumors developed after implantation of PIN-SCs, shPIN-SCs or IL-30shPIN-SCs in WT mice. ANOVA: *p* < 0.01. Tukey’s HSD test: *p* < 0.01 versus both controls. **f** H&E stained sections of lung metastasis spontaneously developed in WT and in IL-30KO mice bearing shPIN-SC or IL-30shPIN-SC tumors. Magnification: × 400. Scale bars: 30 μm. **g** Percentage of lung metastasis spontaneously developed in WT and in IL-30KO mice bearing shPIN-SC or IL-30shPIN-SC tumors. *Fisher’s exact test: *p* < 0.01 versus shPIN-SC tumors in both WT and IL-30KO mice. **h** Histologic (H&E) and immunohistochemical features of tumors developed after sc implantation of shPIN-SCs or IL-30shPIN-SCs in WT and in IL-30KO mice. Magnification: × 400. Scale bars: 30 μm

The hypothesis of a patho-biological role for host–derived IL-30 in tumor progression was tested by using p28 conditional knockout, EIIa-p28^f/f^ mice (C57BL/6 J background), hereinafter referred to as IL-30KO mice. These mice were sc injected with PC-SLCs isolated from Prostatic Intraepithelial Neoplasia (PIN), spontaneously arisen in B6 TRAMP mice (hemizygous for the rat probasin *Pb*-SV40gp6 large T antigen transgene in a C57BL/6 J background, refs [12–14]. These cells, namely PIN-SCs [8] are characterized by a Sca-1^+^CD133^+^CD44^hi^α2β1^hi^ phenotype and lack of CD45 and CD31 markers [13], as validated by flow cytometry (Fig. 1A), to exclude the use of differentiated cells. PIN-SCs have endless self-renewal ability, PC-SLC-specific molecular signature [14] and tumor generating capability at a very low cell number in immune-competent host [8]. Furthermore, they express both IL-30 receptor (R) chains, gp130 and IL-6Rα [8], and constitutively express and release IL-30, whereas they do not produce neither EBI3, nor the IL-27 heterodimer [8].

Eighteen days after their subcutaneous sc implantation, PIN-SCs (1 × 10^5^ cells for a 100% tumor take; ref. [8] gave rise to tumors that grew significantly slower in IL-30KO (IL-30^+/-^tumors) than in WT (IL-30^+/+^tumors) mice. Their mean volume (MTV) was significantly lower than that of tumors developed in WT mice (MTV; 789.76 ± 295.17 versus 1629.56 ± 132.69 mm^3^; student’s t-test: *p* < 0.0001; Fig. 1B).

Since the suppression of IL-30 production by PIN-SCs, via shRNA silencing (IL-30shPIN-SC cells), substantially hindered tumor onset and progression in congenic host [8], we wondered whether the concomitant lack of host-derived IL-30 might strengthen these effects.

Implantation of IL-30shPIN-SCs in IL-30KO mice gave rise to small IL-30^-/-^tumors with a MTV (91.22 ± 6.75 mm^3^) that was not only considerably lower than that of tumors developed in WT mice (IL-30^-/+^tumors) (MTV: 464.39 ± 196.41 mm^3^) (Student’s t-test: *p* < 0.001) (Fig. 1C), but that was also drastically reduced (*p < 0.01*) when compared to the MTV of PIN-SC- and shPIN-SC-tumors (931.77 ± 120.70 mm^3^ and 1007.43 ± 197.69 mm^3^, respectively) developed in IL-30KO mice (Fig. 1D). Of note, the survival of IL-30KO mice bearing IL-30shPIN-SC tumors was considerably longer than that of WT mice bearing IL-30shPIN-SC tumors (64 versus 51 days) (Log-rank test: *p* = 0.047430) (Fig. 1C). The latter, in turn, survived longer than WT mice bearing PIN-SC or shPIN-SC tumors, which had to be sacrificed within 36 days (Chi-square test: *p* < 0.0001) (Fig.1E). Thus, the lack of host immune cell derived IL-30 may improve the effects of IL-30 silencing in PC-SLCs on tumor growth and host behavior.

To assess the consequences of the lack of host- and PC-SLC- derived IL-30 in tumor progression, we next looked at the development of spontaneous metastases in both WT and IL-30KO mice, bearing IL-30-silenced or control tumors (mice were sacrificed when the primary tumors reached similar average volumes; 795 mm^3^, ANOVA: *p* = 0.775049).

Autopsy and histopathological examinations of the different organs (liver, lung and spleen) confirmed that, as observed when orthotopically implanted into the prostate [8], PIN-SCs primarily metastasized to the lungs (Fig. 1F). However, when silenced for IL-30 gene their metastatic capacity was greatly reduced. Indeed, 73% (22/30) of WT mice bearing IL-30shPIN-SC tumors were metastasis-free, whereas only 33% (10/30) of mice bearing wild type PIN-SC- or control shPIN-SC tumors were found metastasis-free at the time when the primary tumor had reached the same volume (Fisher’s exact test: *p* = 0.004) (Fig. 1G). The percentage of metastasis-free mice reached 80% (24/30) in IL-30KO mice bearing IL-30shPIN-SC tumors, whereas only 37% (11/30) of IL-30KO mice bearing control tumors were metastasis-free (Fisher’s exact test: *p* = 0.0006) (Fig. 1G). Therefore, lung metastasis is primarily modulated by the inhibition of IL-30 in PC-SLCs than in the host environment.

### IL-30^−/−^tumors display a poor vascularization, frequent apoptotic events associated with a prominent CD4^+^T cell infiltrate and lack of CD4^+^Foxp3^+^Treg cells

To uncover the mechanisms underlying the slow growth of control tumors in IL-30KO mice and the anti-tumor efficacy of the IL-30 double blockade (in both PC-SLCs and host environment), we first assessed in vivo the tumor viability and immune cell infiltrate.

Although histological aspects of PIN-SC tumors grown in IL-30KO mice were similar to those of PIN-SC tumors developed in WT mice (small epithelioid to round cells, frequent mitosis and a rich vascularity), some apoptotic features emerged (cell shrinkage and nuclear condensation and fragmentation). By contrast, IL-30^−/−^tumors, in addition to a worsening of the ischemic-coagulative necrosis, typical of a vascular deficiency, showed frequent apoptotic figures (Fig. 1H).

Immunohistochemistry confirmed the severe impairment (*p* < 0.01) of the vascular supply in IL-30^−/−^tumors, which was significantly (*p* < 0.01) reduced compared to the already poor vascularization observed in IL-30-silenced tumors grown in WT mice (*p* < 0.01). Consistently, the vascularization of control tumors was prominent and similar in IL-30KO and WT mice (Table 1 and Fig. 1H).

**Table 1:** Immunohistochemical features of IL-30-silenced tumors developed in WT and IL-30KO mice

	shPIN-SC						IL-30shPIN-SC						ANOVA *p* value^b^
	WT			IL-30KO			WT			IL-30KO			
Microvessel density^a^	17.0	±	4.2	15.5	±	4.4	7.3	±	4.0^d^	3.0	±	2.3^c^	< 0.0001
Sca-1^+^ (%)^a^	52.7	±	9.7	48.8	±	8.3	26.5	±	6.6^d^	20.3	±	6.9^d^	< 0.0001
Proliferation Index (%)^a^	68.9	±	8.4	65.0	±	7.5	35.8	±	6.0^d^	36.5	±	7.0^d^	< 0.0001
Apoptotic Index (%)^a^	5.8	±	3.0	16.9	±	4.4^e,f^	10.2	±	4.7	28.2	±	5.6^c^	< 0.0001
Caspase-3 (%)^a^	2.1	±	1.5	14.6	±	2.5^e,f^	3.3	±	1.9	21.8	±	5.0^c^	< 0.0001
Perforin (%)^a^	2.0	±	1.9	12.3	±	3.7^e,f^	2.8	±	2.1	30.0	±	6.3^c^	< 0.0001

^a^Microvessel density and the percentage of immunostained cells were assessed by light microscopy, at × 400 in an 85431.59 μm^2^ field, with Qwin image analysis software (version 2.7). Results are expressed as mean ± SD of CD31 positive microvessels per field (microvessel density), or mean percentage of positive cells/number of total cells (Sca-1, PCNA, TUNEL, caspase-3, perforin)

^b^One-way ANOVA for comparisons between all groups

^*c*^*p* < 0.01 Tukey’s HSD test compared with IL-30shPIN-SC in WT mice and control tumors in WT and KO mice

^*d*^*p* < 0.01 Tukey’s HSD test compared with control tumors in WT and KO mice

^*e*^*p* < 0.01 Tukey’s HSD test compared with control tumors in WT mice

^*f*^*p* < 0.01 Tukey’s HSD test compared with IL-30shPIN-SC in WT mice

Both the frequency of Sca-1 positivity, which characterizes the stem phenotype, and of PCNA positivity, which marks proliferation, were substantially (*p* < 0.01) reduced in IL-30-silenced tumors when compared with controls, regardless of whether the tumor hosts were WT or IL-30KO (Table 1 and Additional file 2: Figure S1).

Interestingly, the apoptotic events, already evident from the histology, were confirmed by the TUNEL assay*,* which showed that apoptotic cells were more frequent (*p* < 0.01) in control tumors developed in IL-30KO mice than in those from WT mice. Furthermore, apoptosis was higher in IL-30^−/−^tumors, when compared with control tumors from both IL-30KO and WT mice (*p* < 0.01), but also when compared with IL-30-silenced tumors from WT mice (*p* < 0.01) (Table 1 and Fig. 1H), thus, suggesting the involvement of host-derived IL-30 in regulating cancer cell apoptosis, whereas proliferation and stemness remained unaltered.

**Fig. 2:**
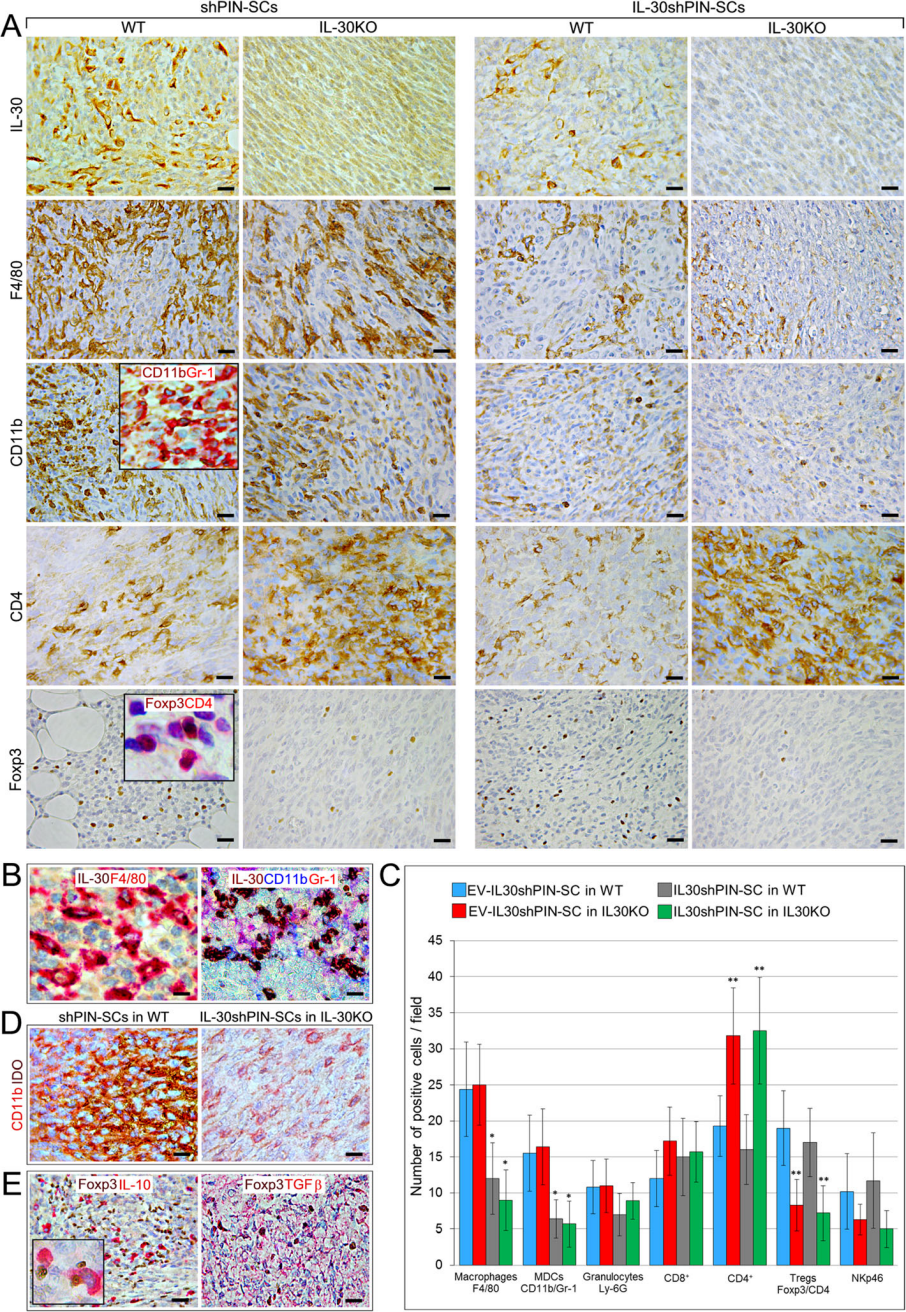
Immunopathological profile of tumors developed after sc. implantation of IL-30-silenced PIN-SCs in WT and IL-30KO mice. **a** Immunohistochemical features of IL-30shPIN-SC and shPIN-SC tumors developed in IL-30KO and in WT mice. Magnification: X400. Scale bars: 30 μm. The insets show double staining for CD11b (brown) and Gr-1 (red) (X630) and double staining for Foxp3 (brown) and CD4 (red) (X1000). **b** In shPIN-SC tumors grown in WT mice, double staining reveals that IL-30 (brown) co-localize with F4/80^+^ macrophages (red), whereas triple staining reveals that IL-30 (brown) also co-localizes with CD11b (blue) and Gr-1 (red), both markers for MDCs. Magnification: × 630. Scale bars: 20 μm. **c** Immune cell counts in IL-30shPIN-SC and control shPIN-SC tumors developed in WT and in IL-30/p28^f/f^ mice. Results are expressed as mean ± SD of positive cells/field evaluated at X400 (0.180 mm^2^ field) by immunohistochemistry. ANOVA: p < 0.01. *p < 0.01, Tukey HSD Test compared with shPIN-SCs in WT or IL-30KO mice. **p < 0.01, Tukey HSD Test compared with shPIN-SCs or IL-30shPIN-SCs in WT mice. **d** Double immunostainings of shPIN-SC tumors developed in WT mice and IL-30shPIN-SC tumors developed in IL-30KO mice (IL-30^−/−^tumors) reveal a strong expression of IDO (brown), which mostly co-localize with CD11b cells (red), in IL-30^+/+^tumors; whereas it is scanty in IL-30^−/−^ tumors. Magnification: × 630. Scale bars: 20 μm. **e** Double immunostainings of shPIN-SC tumors developed in WT mice reveal that IL-10 and TGFβ (both in red) mostly co-localize with Foxp3^+^ cells (brown). Magnification: X400. Scale bars: 30 μm. The inset shows the double staining for Foxp3 (brown) and IL-10 (red): X1000

In the tumor microenvironment (TME), IL-30 expression was evident in cancer cells forming shPIN-SC tumors (in both WT and KO mice), but also in CD11b^+^Gr-1^+^ myeloid-derived cells (MDCs) and macrophages which infiltrated shPIN-SC tumors and, to a lesser extent, IL-30shPIN-SC tumors growing in WT mice, whereas, it was lacking in IL-30-silenced tumors developed IL-30KO mice (Fig. 2A and B).

Analyses of the intra-tumoral inflammatory infiltrate revealed a drastic reduction (ANOVA: *p* < 0.0001, Tukey’s HSD test: *p* < 0.01) of F4/80^+^macrophages and MDCs in IL-30shPIN-SC tumors, both in those grown in WT and in IL-30KO mice compared to control tumors in both strains, whereas the Ly-6G^+^granulocyte content remained substantially unchanged (Fig. 2A, C). The reduced MDC infiltrate was accompanied by an evident decrease in the expression of Indoleamine 2,3-Dioxygenase (IDO) (among the tested immunosuppressive mediators, Arg1, iNOS, NOS2, bFGF) as revealed by double immunostainings (Fig. 2A, D). The number of NKp46^+^ cells showed only a downward trend in tumors grown in IL-30KO mice, whereas, within the lymphocyte population, CD4^+^cells were clearly increased in both control and IL-30-silenced tumors of IL-30KO mice, when compared to tumors of WT mice (ANOVA: *p* < 0.0001, Tukey’s HSD test: *p* < 0.01). CD4^+^Foxp3^+^Tregs were almost absent in control and IL-30-silenced tumors of IL-30KO mice, while they were well represented and evenly distributed in both control and IL-30-silenced tumors of WT mice (ANOVA: *p* < 0.0001, Tukey’s HSD test: *p* < 0.01) (Fig. 2A, C).

Double immunostainings revealed that in both control and IL-30-silenced tumors developed in WT mice, Foxp3^+^cells co-localized with the expression of immunosuppressive cytokines TGFβ and IL-10, which was also frequently observed in macrophage-like cells close to Foxp3^+^cells (Fig. 2E).

These data, which suggested that host-derived IL-30 conditions the intra-tumoral content of CD4^+^Foxp3^+^Tregs and CD4^+^T lymphocytes, led our investigation on their arrangement and functional status within the TME, and within the lymphoid tissue of KO mice.

### CD3^+^T lymphocytes, mostly CD4^+^, infiltrating IL-30^−/−^tumors express cytotoxic molecules and are close to caspase-3^+^ apoptotic cancer cells

**Fig. 3:**
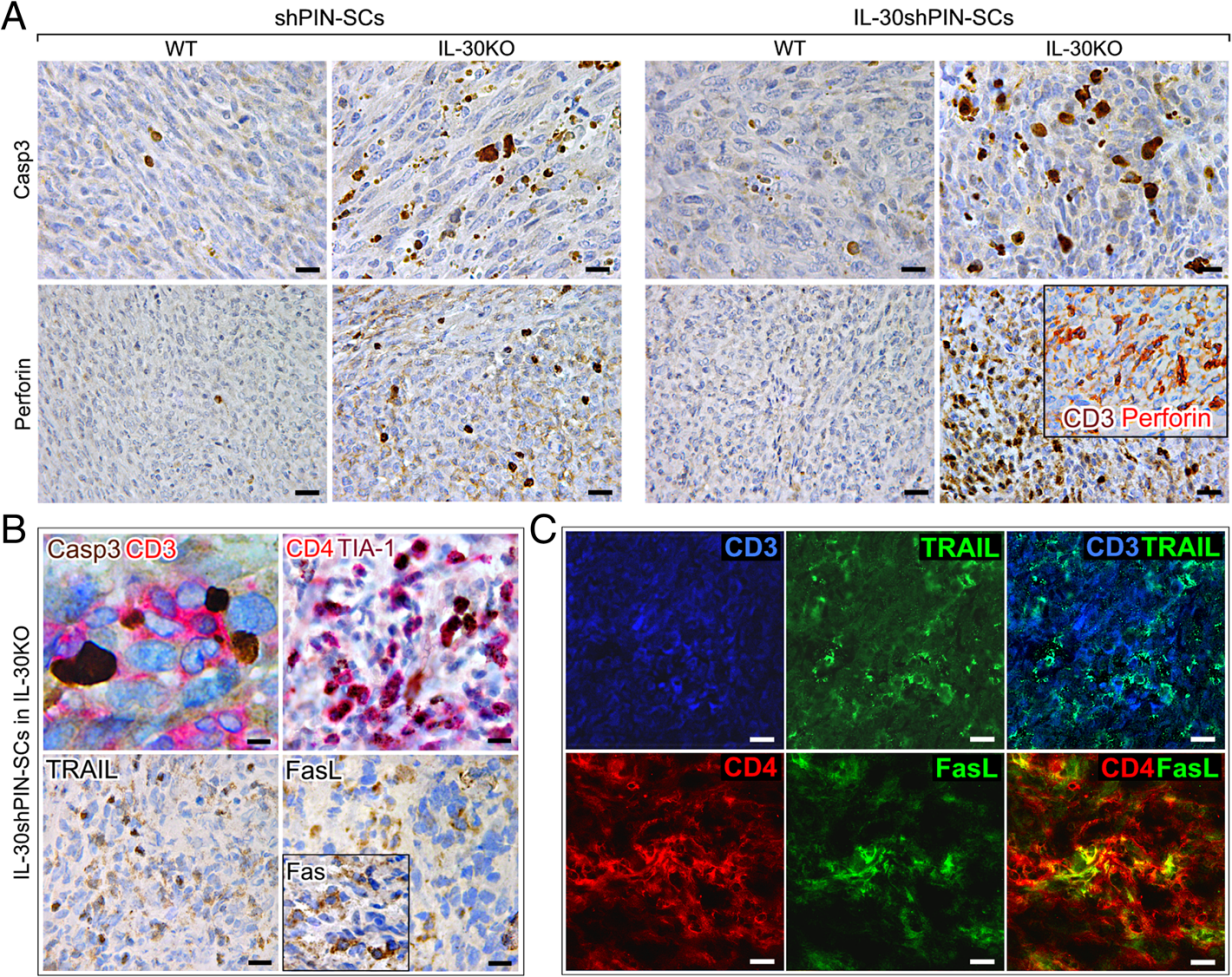
Immunohistochemical detection of apoptosis-related proteins and CTL cytotoxic molecules in tumors that developed after sc implantation of IL-30-silenced PIN-SCs in IL-30KO mice. **a** Active caspase-3 and perforin immunostainings in IL-30shPIN-SC and shPIN-SC tumors developed in IL-30KO and in WT mice. Magnification: × 630 (top images) X400 (bottom images). Scale bars: 20 μm (top images), 30 μm (bottom images). In the inset, the double staining shows perforin (red) co-localization with CD3^+^ cells (brown) (× 400). **b** Double staining of IL-30^−/−^tumors showed CD3^+^T cells (red) in close contact with caspase-3^+^ neoplastic cells (brown). Magnification: X1000. Scale bar: 10 μm. Double staining of IL-30^−/−^tumors also shows that CD4^+^ cells (red) mostly express TIA-1 (brown). Immunohistochemical detection of TRAIL, FasL and Fas (inset) in IL-30^−/−^tumors. Magnification: × 400. Scale bars: 30 μm. **c** Double immunofluorescent stainings of IL-30^−/−^tumors reveal in light blue the co-localization of TRAIL (green) and CD3^+^T cells (blue) and in yellow the co-localization of FasL (green) and CD4^+^T cells (red). Magnification: X630. Scale bars: 20 μm

The defective recruitment of MDCs was the hallmark of IL-30-silenced tumors, regardless of the mouse strain, while the absence of Tregs and the significant CD4^+^T cell infiltrate, configured all the tumors (control and IL-30-silenced) arising in IL-30KO mice. The apoptotic events, that prevailed in these tumors, were confirmed by immunostainings for activated caspase-3, whose expression is typically associated with cytotoxic T lymphocyte (CTL)-mediated cytotoxicity [21]. Caspase-3^+^ neoplastic cells were more numerous (*p* < 0.01) in control tumors developed in KO than in WT mice. Furthermore, they were significantly more frequent in IL-30^−/−^tumors compared to control tumors in both KO and WT mice, and compared to IL-30-silenced tumors in WT mice (*p* < 0.01) (Fig. 3A and Table 1).

The double immunohistochemistry revealed that, in control tumors developed in IL-30KO and, especially, in IL-30^−/−^tumors, the caspase-3^+^ cancer cells were in close contact with, and frequently embraced by, CD3^+^T lymphocytes (Fig. 3B), thus suggesting their cytotoxic effector function. Among these, the CD4^+^ cells, which was the prevalent population, mostly expressed the cytotoxic granule-associated RNA binding protein TIA-1 (Fig. 3B).

Assessment of CTL-associated cytotoxic molecules, granzyme B and perforin, revealed that expression, specifically of the latter, was strong in IL-30^−/−^tumors, distinct in control tumors grown in KO mice, and absent in IL-30-silenced and control tumors grown in WT mice (*p* < 0.01) (Fig. 3A and Table 1). Double stainings also revealed perforin co-localization with CD3^+^T cells (Fig. 3A, inset).

Expression of CTL-associated molecules, TNF-related apoptosis-inducing ligand (TRAIL) and FasL, was detected in control tumors grown in IL-30KO mice and, especially, in IL-30^−/−^tumors, whereas expression of the Fas/CD95 death receptor concerned most of neoplastic cells, regardless of whether they were silenced or not for IL-30, and regardless of the mouse strain they were implanted in (Fig. 3B, inset), thus suggesting a potential sensitivity of PIN-SC tumors to FasL-mediated apoptosis.

Immunofluorescence and confocal analyses revealed that, in tumors grown in IL-30KO mice, many of the CD3^+^T cells expressed TRAIL (Fig. 3C) and that FasL expression mostly co-localized with tumor-infiltrating CD4^+^T cells (Fig.3C).

### The spleen of IL-30KO mice lacks the expansion of CD4^+^CD25^+^Foxp3^+^ Tregs and expression of IL-10, whereas IFNγ and IL-12 expression increases following PC-SLC implantation

The distinctive features of the immune cell infiltrate of both IL-30-silenced and control tumors developed in IL-30KO mice, which were enriched with CD4^+^T lymphocytes and lacked Tregs, prompted us to characterize the lymphoid tissue in this mouse strain. As expected, the production of IL-30 was missing in the spleen of KO mice, while it was scanty, but detectable, in the reticular framework of marginal zone macrophages and dendritic-like cells in the spleen of WT mice (Additional file 2: Figure S2). IL-30 production appeared substantially unchanged in the spleen of WT mice following tumor cell implantation.

**Fig. 4:**
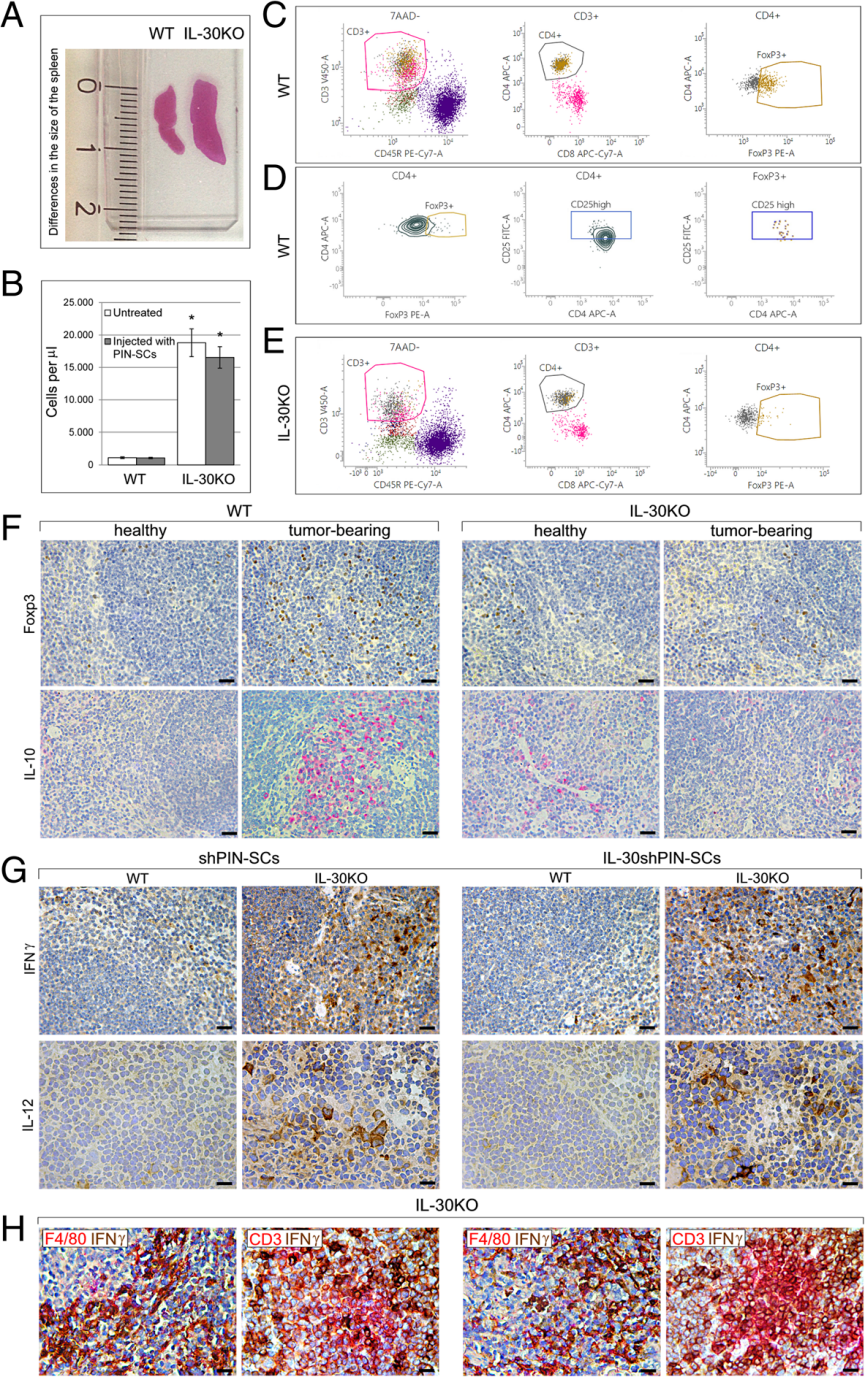
Histopathological and cytofluorimetric aspects of the spleen of IL-30KO mice. **a** H&E stained sections of the spleens obtained from untreated WT and IL-30KO mice showing differences in macroscopic appearance and in size. **b** Total number of cells in the spleens from WT or IL-30KO mice, injected or not with 1X10^5^ PIN-SCs. Results are reported as mean ± SD of viable cells evaluated by flow cytometry, using 7-amino-actinomycin D (7-AAD) staining. **c** and **d** illustrate flow cytometry analysis of Treg cells in one representative spleen sample from a WT mouse, injected with PIN-SCs. **c**. After exclusion of dead cells (7-AAD-positive), CD3 + CD4 + CD45R- nucleated cells were gated and analyzed for Foxp3. **d.** Most of the CD4^+^Foxp3^+^ population showed high CD25 expression. Isotype controls were used to assess the background. Experiments were performed at least in triplicate. **e** Flow cytometry analysis of Tregs in one representative spleen sample from an IL-30KO mouse, injected with PIN-SCs. After exclusion of dead cells (7-AAD-positive), CD3^+^CD4^+^CD45R^−^ nucleated cells were gated and analyzed for Foxp3. Experiments were performed at least in triplicate. **f** Immunohistochemical features of spleens obtained from healthy and (PIN-SC) tumor-bearing WT and IL-30KO mice. Results obtained from mice bearing control shPIN-SC tumors are not different from those obtained in mice bearing PIN-SC tumors. Magnification: × 400. Scale bars: 30 μm. **g** Immunohistochemical features of spleens obtained from WT and IL-30KO mice bearing shPIN-SC or IL-30shPIN-SC tumors. Results obtained from WT and KO mice bearing control shPIN-SC tumors are not different from those obtained in the same mouse strain bearing PIN-SC tumors. Magnification: × 400 (top); × 630 (bottom). Scale bars: 30 μm (top); 20 μm (bottom). **h** Double immunohistochemistry reveals that in the spleen of IL-30KO mice bearing shPIN-SC or IL-30shPIN-SC tumor, IFNγ (brown) mostly co-localizes with F4/80^+^ macrophages (red) and, to a lesser extent with CD3^+^T cells (red). Magnification: X400.Scale bars: 30 μm

Macroscopic examination of the spleens of IL-30KO mice, revealed that they were more bulky and significantly heavier than spleens of WT mice (0.12 ± 0.02 g versus 0.10 ± 0.01 g Student’s *t*-test: *p* = 0.044) (Fig. 4A). A similar difference was also evident in tumor-bearing mice (0.11 ± 0.01 g versus 0.08 ± 0.01 g. Student’s *t*-test: *p* < 0.00001).

Flow cytometry analysis showed that the spleens from IL-30KO mice, both the untreated and those bearing tumors, had a greater cellularity than the spleens of WT mice (ANOVA: *p* < 0.0001, Tukey’s HSD test: *p* < 0.01) (Fig. 4B), but maintained a similar percentage of T cell (CD3, CD8a, CD4) and B cell (CD45R) content. After tumor cell implantation, unlike in WT mice, the spleen of IL-30KO mice lacked the expansion of the CD4^+^CD25^hi^Foxp3^+^ regulatory T (Treg) cell population (IL-30KO mice: 5.64 ± 2.23% versus WT mice: 19.33 ± 5.60% of the total number of CD4^+^ cells. ANOVA: *p* < 0.0001, Tukey’s HSD test: *p* < 0.01) as shown in Fig. 4C, D, E.

Immunohistochemistry corroborated the cytofluorimetric data by detecting an expansion of Foxp3^+^ cells, mostly distributed in the T cell areas, in the spleen of tumor-bearing WT mice (18.8 ± 4.3%) versus both healthy WT (7.1 ± 4.2%) and KO (7.5 ± 3.6%) mice and versus tumor-bearing IL-30KO mice (9.2 ± 4.0%) (Tukey’s HSD test: p < 0.01), regardless of IL-30 silencing in the implanted tumor cells (Fig. 4F).

In WT mice, the Treg cell rich zones showed a distinct expression of IL-10 (Fig. 4F), while IL-12 and IFNγ were substantially absent. On the contrary, in the spleen of IL-30KO mice bearing IL-30shPIN-SC or shPIN-SC tumors, the network of macrophage- and dendritic-like cells clearly expressed both IL-12 and IFNγ (Fig. 4G). Double staining clearly demonstrated IFNγ co-localization with F4/80^+^ macrophages and CD3^+^T cells (Fig. 4H).

### IL-30^neg^PC patients show a significant CD4^+^T and a poor Foxp3^+^ cell infiltrate in their surgical samples and have a lower incidence of BCR than IL-30^pos^PC patients

The peculiar immuno-phenotypical profile associated with the drastic growth inhibition of IL-30-silenced tumor in IL-30KO mice (IL-30^−/−^tumors), which survived much longer than WT mice bearing IL-30 expressing tumor (64 *versus* 36 days, Chi-square test: *p* < 0.0001), prompted us to assess whether it was consistent with the histopathological features of clinical samples and patient outcome.

We previously observed that IL-30 expression typically involved high grade and stage of disease [6], therefore, we analyzed (n.112) PC samples, classified as Gleason score 8 to 10 (high-grade tumors), from patients with stage III disease (high-risk, clinically localized PC), a class of patients whose therapeutic management is still debated and needs to be improved [16]. After staining for IL-30, we applied the evaluation criteria previously described in ref. [6], and selected PC samples with or without IL-30 expression, in both tumor cells and infiltrating leukocytes (ILK) (IL-30^Pos^PC; n.25, and IL-30^Neg^PC; n.59, respectively).

**Fig. 5:**
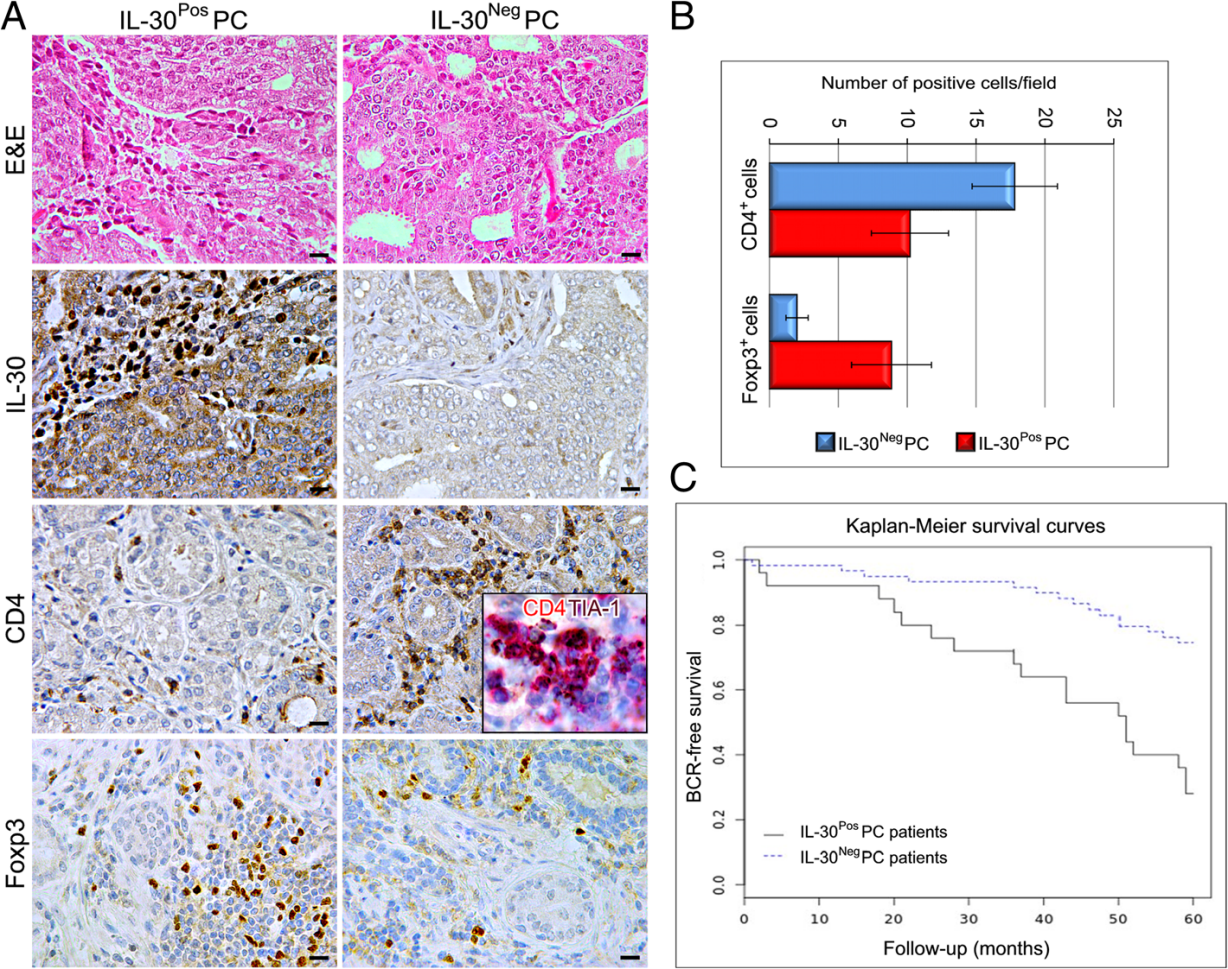
Immunophenotypical aspects of high grade and stage IL-30^Pos^ and IL-30^Neg^ PC and prognostic evaluations. **a** Histologic (H&E) and immunohistochemical features of high grade and stage IL-30^Neg^PC and IL-30^Pos^PC. Magnification: X400. Scale bars: 30 μm. The inset shows TIA-1 (brown) co-localization with CD4 (red) (X1000). **b** Immune cell counts in high grade and stage IL-30^Neg^PC and IL-30^Pos^PC. **c** Kaplan–Meier estimates of BCR for stage III patients with Gleason score 8–10, classified as IL-30^Pos^PC (n.25) and 30^Neg^PC (n.59)

Immunohistochemistry revealed that the CD4^+^T cell infiltrate was considerable (18.8 ± 3.1) in IL-30^Neg^PC samples, whereas the Foxp3^+^ cell content was scanty to absent (2.0 ± 0.8) when compared with IL-30^Pos^PC samples (CD4^+^cells: 10.20 ± 2.8; Foxp3^+^cells: 9.5 ± 2.9; Student’s t-test: *p* < 0.001) (Fig. 5A, B). Furthermore, lymphocytes infiltrating IL-30^Neg^PC mostly expressed the activation marker TIA-1 [22] (inset in Fig. 5A).

Kaplan-Meier survival curves showed a significantly shorter Disease-Free Survival for patients with IL-30^Pos^PC (18 out of 25 patients with biochemical recurrence, BCR, 72%) versus those with IL-30^Neg^PC (15 out of 59 patients with BCR, 25%) (Log-rank test: *p* = 0.000022) (Fig. 5C).

## Discussion

Despite immunotherapy has revealed to be promising for the treatment of advanced tumors [23], ongoing trials for PC have achieved poor clinical responses [24, 25]. Overcoming immunosuppression generated by the aberrant tumor-host relationship and breaking resistance to current immunotherapeutic strategies is a major challenge for oncology.

In an attempt to define the molecular mediators of the PC – host interactions, we identified the expression of the cytokine IL-30, also known as IL-27p28, which we recently investigated throughout the natural history of prostate cancer [6]. Absent in normal prostatic epithelia, the production of IL-30, which in the early stages of the disease is confined to the rare PC-SLCs, typically characterizes the poorly differentiated, high-grade PC, possibly due to the role of this very small cell population as a major component and driver of key processes in cancer progression, such as tumor growth, recurrence and metastasis [26]. Furthermore, tumor infiltrating leukocytes, most of which MDCs, are main source of the cytokine [6]. This occurs primarily in advanced stages of PC [6, 7], which are characterized by intra-tumoral immature MDCs that promote tumor vascularization [27] and suppress DC functions [28] and T cell activation [29, 30].

We have previously shown that IL-30 boosts cancer cell expression of CCL4, CSF-2, CSF-3, CXCL1, CXCL2 and PTGS2, which along with IL-1β, IL-6 and TNFα, promotes MDC accumulation and immunosuppressive activity [8, 31], whereas IL-30 silencing in PC-SLCs, reduces the tumor infiltration of MDCs, depletes the vascular supply and prevents or delays PC onset and progression [8]. Here, we provide evidence of the synergistic effect of IL-30 knockdown, in both PC-SLCs and host environment, in hampering tumor growth and progression and improving host survival.

Deletion of IL-27p28 alleles makes the IL-27p28^f/f^ conditional KO mice, used in our study, unable to produce IL-30, as confirmed by the complete lack of production of the cytokine in the splenic tissue and in the TME. Mainly produced by activated APCs [3, 32], IL-30 has been recognized as a self-standing cytokine [3], which acts by recruiting a gp130 homodimer and signals via IL-6Rα [33]. Alternatively, it may be co-expressed with EBI3 to form the heterodimeric IL-27, which engages the WSX-1/gp130 receptor complex [3], or may combine with cytokine-like factor (CLF) to form a functional complex which, as well as IL-30, has been described to bind to IL-6Rα and gp130 [34]. Studies carried out so far, using IL-30KO mice, suggest that the lack of host IL-30 increases the susceptibility to liver injury, by boosting IFNγ production by CD4^+^T cells [15], increases the sensitivity to LPS-induced sepsis, through the inhibition of IL-10 and up-regulation of IFNγ production by Natural Killer–like T cells [35]. Our results are consistent with previous findings and demonstrate, for the first time, that the lack of leukocyte-derived IL-30 hinders CD4^+^CD25^+^Foxp3^+^Treg expansion and immunosuppressive cytokine production in the lymphoid tissues and in the TME. When combined with IL-30 knockdown in PC-SLCs, it significantly affects tumor growth and host survival.

It should be emphasized that IL-30 silencing in PC-SLCs greatly reduces the intra-tumoral recruitment of leukocytes, particularly macrophages and MDCs, which are potential source of IL-30, thus determining a reduction of IL-30 within the TME, and mimicking the lack of IL-30-producing leukocytes, which characterize IL-30KO mice.

If the altered immunological equilibrium resulting from the absence of IL-30/IL-27p28 production, can be ascribed to IL-30 in itself or to the coexisting lack of IL-27 or other p28-containing molecular complexes, remains a critical issue.

The lack of host-derived IL-30 not only prevents the pathophysiological expansion of Tregs in the spleen, after PC-SLC engraftment, but also shapes the TME by abolishing the influx of IL-10^+^TGFβ^+^Tregs, while it promotes that of CD4^+^T lymphocytes. The prevention of intra-tumoral immunosuppressive IDO^+^MDC infiltrate, due to IL-30 silencing in PC-SLCs, likely boosts the cytotoxic and tumoricidal activity of T lymphocytes, as suggested by the frequency of caspase-3^+^ apoptotic cancer cells close to CD3^+^T lymphocytes, which express cytotoxic molecules perforin, TRAIL or FasL.

Besides improving the efficacy of tumor-reactive CD8^+^T cells [36, 37], the CD4^+^T cells have been recognized with a CTL activity in both tumor models [38] and human anti-tumor responses [39]. Expansion of effector perforin^+^FasL^+^CD4^+^T cells has been described in cancer patients, during neo-adjuvant chemotherapy, in proportion to clinical response [40]. Besides killing directly MHC class II positive tumor cells, CD4^+^ T lymphocytes can indirectly eliminate cancer cells lacking of MHC class II [38], which is the most frequent condition in tumors, including PC [40], and in our PC-SLCs. These cells are equipped with MHC class I and can express MHC class II, following IFNγ treatment [13]. After tumor cell challenge, IL-30 conditional KO mice showed an imbalance towards a Th1-type immune response, as revealed by IFNγ and IL-12 expression in splenic lymphocytes and macrophages. The question of whether these cytokines can promote CD4^+^CTL activity [41–43] or be involved in the inhibition of tumor angiogenesis [44], remains to be explored. Indeed, in IL-30^-/+^ and IL-30^−/−^tumors, the F4/80 macrophage and IDO^+^CD11b^+^Gr-1^+^ myeloid cell content was somewhat depleted, which in itself compromises tumor angiogenesis and alleviates suppression of T cell activity [30]. Only in IL-30^−/−^tumors, the significant influx of CD4^+^T cells and the lack of Treg suppressive functions, both dependent on IL-30KO immunophenotype, coexist with the scanty intra-tumoral MDC infiltrate determined by the silencing of IL-30 in PC-SLCs. Killing of PC-SLCs by MHC class I-restricted CD3^+^CD8^+^T cells, involving TRAIL-mediated apoptosis or perforin-mediated lysis of tumor cells [45], may not be ruled out, although CD4^+^ T cells were consistently more represented than CD8^+^ cells and were the prevalent immune cell population in tumors grown in IL-30KO mice.

Death ligands TRAIL and FasL belong to a subgroup of the TNF superfamily, which share a TNF homology domain and induce apoptosis by binding, as trimers, to the corresponding death domain containing receptors. TRAIL can bind two apoptosis-inducing receptors TRAIL-R1 (DR4) and TRAIL-R2 (DR5) [46], whereas FasL binds to CD95/Fas receptor expressed by the majority of cancer cells in PIN-SC tumors.

Active caspase-3, which is widely expressed in IL-30^−/−^tumors as well as TUNEL-positive staining, is involved in the apoptotic signaling cascades of both TRAIL and FasL [46, 47]. Apoptotic cell death is a hallmark of tumors developing in IL-30KO mice. In IL-30^−/−^tumors, apoptotic events occurred together with ischemic-coagulative necrosis associated with an impaired vascularization. Tumor necrosis should release high amounts of tumor-derived antigens to be taken up by APCs and promote CD4^+^T cell activation and an enhanced antitumor response [48].

The absence of IL-30 in the host environment is not sufficient to prevent metastases, but it improves the protective effect of IL-30 silencing in PC-SLCs increasing the percentage of metastasis-free mice from 73% (of WT mice bearing IL-30-silenced tumors) to 80% (of IL-30KO mice bearing IL-30-silenced tumors).

The data from tumor-bearing IL-30KO mice led our immunophenotypical analyses of PC tissues from patients with high-grade, locally advanced disease, for which management guidelines and prognostic criteria are highly debated [16]. We focused on patients bearing IL-30^Pos^ or IL-30^Neg^ tumors, i.e. with or without IL-30 expression in both cancer and ILK, which mimicked the IL-30^+/+^tumors grown in wild-type mice, and IL-30^−/−^tumors from IL-30KO mice, respectively. As described in murine tumor tissues, in IL-30^Neg^PC samples, the lack of IL-30 in both cancer and infiltrating leukocytes was associated with a scanty to absent Foxp3^+^Treg cell content and a distinct TIA-1^+^CD4^+^T cell infiltrate. By contrast, IL-30^Pos^PC samples were rich in Foxp3^+^Tregs and lacking in CD4^+^T cells. Most of all, among patients undergoing prostatectomy due to high-grade and locally advanced PC, those with IL-30^Neg^ cancer showed, as revealed by the Kaplan-Meier curve, a longer disease-free survival, thus suggesting the potential value of translating these research findings to the clinical practice.

## Conclusions

Our data provide the proof of concept that targeting IL-30 in both cancer and host environment consistently inhibits tumor growth, ameliorates immune reactivity and reduces the risks of disease recurrence. This study highlights the value of patient-tailored immunotherapy for advanced PC designed to overcome the immunosuppressive PC microenvironment and effectively improve patient outcome.

## Additional files

Additional file 1: **Table S1.** Antibodies used in flow cytometry. **Table S2.** Antibodies used in immunostaining. (DOCX 24 kb)

Additional file 2: **Figure S1.** Sca-1 and PCNA immunostainings in shPIN-SC and IL-30shPIN-SC tumors, developed in WT and IL-30KO mice. **Figure S2.** IL-30 immunostaining in the spleen of WT and IL-30KO mice. (DOCX 775 kb)

## Data Availability

The datasets analyzed during the current study are available from the corresponding author on reasonable request.

## Abbreviations

BCR: Biochemical recurrence
Casp3: Caspase-3
CCL: Chemokine (C-C motif) ligand
CSF: Colony-stimulating factor
CXCL: Chemokine (C-X-C motif) ligand
EIIa-p28^f/f^: IL-27p28 conditional knockout mouse strain
IDO: Indoleamine 2,3-dioxygenase
IFNγ: Interferon gamma
IL: Interleukin
IL-30^+/+^tumors: PIN-SC or shPIN-SC tumors in WT mice
IL-30^-/-^tumors: IL-30shPIN-SC tumors in IL-30KO mice
IL-30^+/-^tumors: PIN-SC or shPIN-SC tumors in IL-30KO mice
IL-30^-/+^tumors: IL-30shPIN-SC tumors in WT mice
ILK: Infiltrating leukocytes
KO: Knockout
MDCs: Myeloid derived cells
MTV: Mean tumor volume
PC: Prostate cancer
PCNA: Proliferating cell nuclear antigen
PC-SLCs: Prostate Cancer Stem-Like Cells
PIN-SCs: Prostatic intraepithelial neoplasia-derived stem-like cells
PTGS2: Prostaglandin-endoperoxide synthase 2
Sca-1: Stem cells antigen-1
shRNA: Short hairpin RNA
TIA-1: Cytotoxic granule-associated RNA binding protein
TME: Tumor microenvironment
TNF: Tumor necrosis factor
TRAIL: TNF-related apoptosis-inducing ligand
Tregs: T regulatory cells
TUNEL: Terminal deoxynucleotidyl transferase dUTP nick end labeling
WT: Wild type
