# Supplementary material for: Targeting Interleukin(IL)-30/IL-27p28 signaling in cancer stem-like cells and host environment synergistically inhibits prostate cancer growth and improves survival
Source: J Immunother Cancer. 2019 Jul 31;7:201. doi: 10.1186/s40425-019-0668-z (PMC6670138; doi:10.1186/s40425-019-0668-z)
Supplement: Supplementary file 1 — Table S1. Antibodies used in flow cytometry. Table S2. Antibodies used in immunostaining. (DOCX 24 kb) [file 40425_2019_668_MOESM1_ESM.docx]

**Table S1.** Antibodies used in flow cytometry.

| **Antibody** | **Clone** | **Format** | **Source** |
| --- | --- | --- | --- |
|  |  |  |  |
| CD3 | 17A2 | BD Horizon™ BV™ 421 | BD Biosciences, Franklin Lakes, NJ, USA |
| CD4 | RM4-5 | APC | “ |
| CD8a | 53-6.7 | APC-H7 | “ |
| CD25 | 7D4 | FITC | “ |
| CD29 (Integrin β1) | HM β1-1 | BD Horizon™ BV™ 605 | “ |
| CD31 | MEC 13.3 | BD Horizon™ BV™ 510 | “ |
| CD44 | IM7 | APC | “ |
| CD45 | 30-F11 | PE-Cy™5 | “ |
| CD45R | RA3-6B2 | PE-Cy™7 | “ |
| CD49b (Integrin α2) | HMα2 | BD Horizon™ BV™ 650 | “ |
| CD49f | GoH3 | BD Horizon™ BV™ 711 | “ |
| CD126 | M5 | PE | “ |
| CD130 | B-R3 | FITC | Abcam, Cambridge, UK |
| CD133 | 13A4 | PE | Thermo Fisher Scientific, Waltham, MA, USA |
| Foxp3 | MF23 | PE | BD Biosciences |
| Sca-1 | D7 | BD Horizon™ BV™ 510 | “ |
|  |  |  |  |

**Table S2.** Antibodies used in immunostaining.

| **Antibody** | **Clone** | **Origin** | **Source** | **Positive control** |
| --- | --- | --- | --- | --- |
| ***Antibodies used on mouse samples*** |  |  |  |  |
| Arg1 |  | Rabbit | Thermo Fisher Scientific | Spleen |
| bFGF |  | Rabbit | Santa Cruz, Dallas, TX, USA | Kidney |
| Casp3 |  | Rabbit | Cell Signaling, Danvers, MA, USA | Spleen |
| CD11b | EPR1344 | Rabbit | Abcam | “ |
| CD3 |  | Rabbit | Agilent, Santa Clara, CA, USA | “ |
| CD31 | SZ31 | Rat | Dianova, Hamburg, D | “ |
| CD4^a^ | YTS191.1.2 | Rat | Merck, Darmstadt, Germany | “ |
| CD8^a^ | YTS169.4 | Rat | Oxford Biotechnology, Kidlington, UK | “ |
| F4/80 | Cl:A3-1 | Rat | Bio-Rad, Hercules, CA, USA | “ |
| Fas |  | Rabbit | Santa Cruz | “ |
| FasL |  | Rabbit | Merck | “ |
| Foxp3 | FJK-16s | Rat | Thermo Fisher Scientific | “ |
| Gr-1 | RB6-8C5 | Rat | BD Biosciences | “ |
| Granzyme B | 11F1 | Mouse | Leica Biosystems, Wetzlar, D | “ |
| IFNγ |  | Rabbit | Abcam | “ |
| IDO | 4B7 | Mouse | Merck | “ |
| IL-10 |  | Rabbit | Abcam | “ |
| IL-12 |  | Goat | PeproTech, London, UK | “ |
| IL-30/IL-27p28 (#AF1834) |  | Goat | R&D, Minneapolis, MN, USA | “ |
| iNOS |  | Rabbit | BD Biosciences | Lung |
| Ly-6G | 1A8 | Rat | BioLegend, San Diego, CA, USA | Spleen |
| NKp46^a^ |  | Rabbit | Biorbyt | “ |
| NOS2 |  | Rabbit | Santa Cruz | Lung |
| PCNA | PC10 | Mouse | Agilent | Spleen |
| Perforin | 5B10 | Mouse | Leica Biosystems | “ |
| Sca-1 (Ly-6A.2/Ly-6E.1) | EPR3355 | Rabbit | Abcam | Kidney |
| TGFβ |  | Goat | R&D | “ |
| TRAIL^a^ |  | Goat | Santa Cruz | Liver |
|  |  |  |  |  |
| ***Antibodies used on human samples*** |  |  |  |  |
| CD4 | MT310 | Mouse | Agilent | Tonsil |
| Foxp3 | mAbcam22510 | Mouse | Abcam | “ |
| IL-30/IL-27p28 |  | Rabbit | “ | “ |
| TIA-1^b^ | TIA-1 | Mouse | “ | Spleen |

^a^Antibodies used on frozen sections.

^b^Antibody also used on mouse tissue sections.
