# Supplementary material for: Targeting Interleukin(IL)-30/IL-27p28 signaling in cancer stem-like cells and host environment synergistically inhibits prostate cancer growth and improves survival
Source: J Immunother Cancer. 2019 Jul 31;7:201. doi: 10.1186/s40425-019-0668-z (PMC6670138; doi:10.1186/s40425-019-0668-z)
Supplement: Supplementary file 2 — Figure S1. Sca-1 and PCNA immunostainings in shPIN-SC and IL-30shPIN-SC tumors, developed in WT and IL-30KO mice. Figure S2. IL-30 immunostaining in the spleen of WT and IL-30KO mice. (DOCX 775 kb) [file 40425_2019_668_MOESM2_ESM.docx]

**
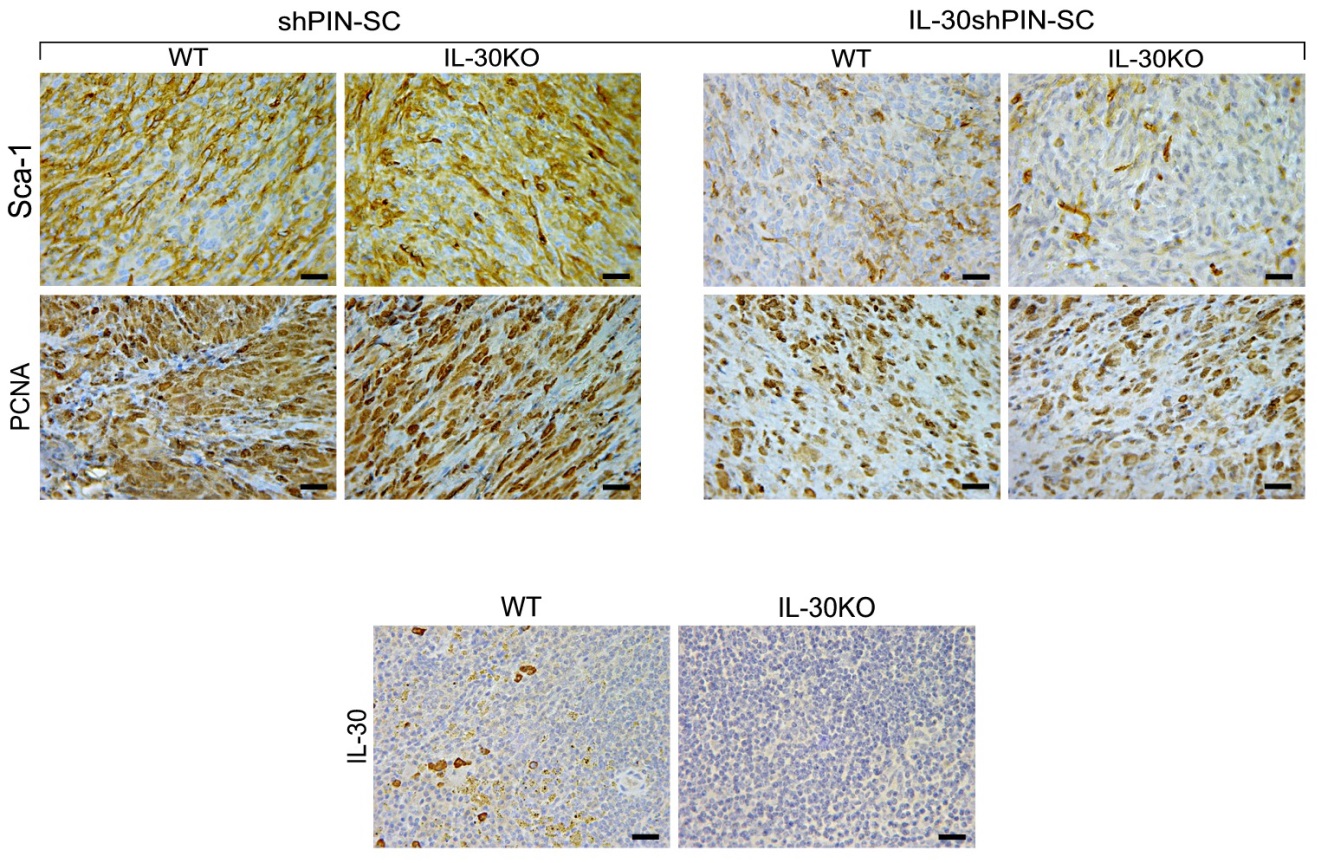
**

**Figure S1.** Sca-1 and PCNA immunostainings in shPIN-SC and IL-30shPIN-SC tumors developed in WT and IL-30KO mice. Magnification: X400. Scale bars: 30 μm.

**
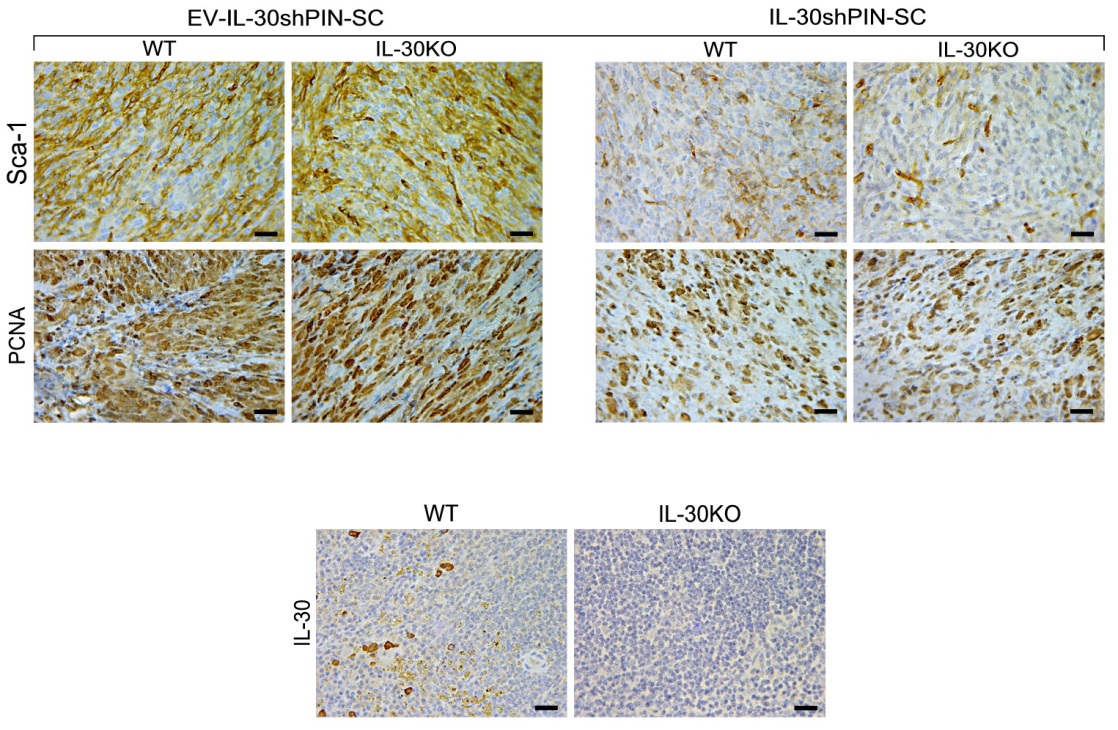
**

**Figure S2.** IL-30 immunostaining in the spleen of WT and IL-30KO mice. Magnification: X400. Scale bars: 30 μm.
